# A Dual Simple Recurrent Network Model for Chunking and Abstract Processes in Sequence Learning

**DOI:** 10.3389/fpsyg.2021.587405

**Published:** 2021-05-04

**Authors:** Lituan Wang, Yangqin Feng, Qiufang Fu, Jianyong Wang, Xunwei Sun, Xiaolan Fu, Lei Zhang, Zhang Yi

**Affiliations:** ^1^Machine Intelligence Laboratory, College of Computer Science, Sichuan University, Chengdu, China; ^2^Institute of High Performance Computing, Agency for Science, Technology and Research, Singapore, Singapore; ^3^State Key Laboratory of Brain and Cognitive Science, Institute of Psychology, Chinese Academy of Sciences, Beijing, China; ^4^Department of Psychology, University of Chinese Academy of Sciences, Beijing, China

**Keywords:** sequence learning, abstract processes, chunking processes, simple recurrent network, dual simple recurrent

## Abstract

Although many studies have provided evidence that abstract knowledge can be acquired in artificial grammar learning, it remains unclear how abstract knowledge can be attained in sequence learning. To address this issue, we proposed a dual simple recurrent network (DSRN) model that includes a surface SRN encoding and predicting the surface properties of stimuli and an abstract SRN encoding and predicting the abstract properties of stimuli. The results of Simulations 1 and 2 showed that the DSRN model can account for learning effects in the serial reaction time (SRT) task under different conditions, and the manipulation of the contribution weight of each SRN accounted for the contribution of conscious and unconscious processes in inclusion and exclusion tests in previous studies. The results of human performance in Simulation 3 provided further evidence that people can implicitly learn both chunking and abstract knowledge in sequence learning, and the results of Simulation 3 confirmed that the DSRN model can account for how people implicitly acquire the two types of knowledge in sequence learning. These findings extend the learning ability of the SRN model and help understand how different types of knowledge can be acquired implicitly in sequence learning.

## Introduction

Implicit learning refers to all unintentional learning, in which the underlying structure of a complex stimulus environment is acquired independently of conscious attempts to do so and the resulting knowledge is difficult to express (Reber, 1989; Seger, 1994; Dienes and Berry, 1997; Shang et al., 2013; Fu et al., 2018). Despite decades of research, it remains controversial whether people can acquire abstract knowledge in implicit learning (Cleeremans, 1994). Some researchers assume that the knowledge acquired in implicit learning is abstract and represents “the structure of the stimuli and their relationships” (Reber, 1989). Early evidence for this “abstractionist” view stemmed primarily from transfer effects in artificial grammar learning (AGL) tasks (Reber, 1967, 1969, 1989; Reber and Lewis, 1977; Altmann et al., 1995; Redington and Chater, 1996; Dienes and Altmann, 1997; Dienes et al., 1999). For example, it was found that participants could transfer grammatical knowledge about memorised strings to novel instances (Mathews, 1990; Cleeremans, 1993; Knowlton and Squire, 1996), even novel instances in different modalities (Altmann et al., 1995; Dienes et al., 1999). However, this “abstractionist” view has been questioned by considerable research (Perruchet and Pacteau, 1990; Brooks and Vokey, 1991; Vokey and Brooks, 1992; Shanks and St John, 1994). For example, it was demonstrated that the transfer effect in AGL was based on the similarity between novel strings and the “whole exemplar” stored in memory during training or explicitly memorised fragments or chunks of materials (Dulany et al., 1984; Mathews et al., 1989; Perruchet and Pacteau, 1990). Gomez (1997) argued that implicit knowledge can be acquired only at the simple level of complexity such as first-order dependencies, whereas other more complex knowledge such as second-order dependencies can only be acquired explicitly.

### What Is Learned in Sequence Learning?

Sequence learning has become one of the most widely used paradigms in research into implicit learning (Cleeremans and Dienes, 2008), in which subjects were asked to complete a serial reaction time (SRT) task (Nissen and Bullemer, 1987). Most studies have focussed on whether second-order dependencies or more complex chunk knowledge can be learned implicitly in sequence learning (Perruchet and Amorim, 1992; Destrebecqz and Cleeremans, 2001, 2003; Wilkinson and Shanks, 2004; Norman et al., 2006, 2007; Fu et al., 2008, 2010; Jiménez et al., 2011; Pasquali et al., 2019). For example, Destrebecqz and Cleeremans (2001) adopted two second-order conditional (SOC) sequences (SOC1 = 3-4-2-3-1-2-1-4-3-2-4-1 and SOC2 = 3-4-1-2-4-3-1-4-2-1-3-2) as training and transfer sequences and found that participants responded to the training SOC sequence much faster than the transfer SOC sequence, indicating learning of the second-order dependencies. Moreover, Destrebecqz and Cleeremans (2001) used an inclusion test and an exclusion test that differed only by instructions to dissociate implicit learning from explicit learning. Under the inclusion test, participants were instructed to generate a sequence that was the same as the training sequence. On the contrary, under the exclusion test, participants were instructed to generate a sequence that was different from the training sequence. They found that participants generated similar numbers of triplets from the training sequence under inclusion and exclusion tests when the response stimulus interval (RSI) was zero, providing important evidence that people can implicitly or unconsciously learn second-order dependencies. However, this crucial finding was not replicated by Wilkinson and Shanks (2004). Fu et al. (2008) replicated the experiments conducted by Destrebecqz and Cleeremans (2001) and Wilkinson and Shanks (2004) separately by manipulating rewards, the amount of noise, and the amount of training, confirming that SOC sequence knowledge can be learned implicitly.

Further, Fu et al. (2008) found that in free-generation tasks, more triplets from training and transfer sequences were generated under the exclusion test in the 6-block group than in the 15-block group, indicating that people acquired knowledge about the structures common to training and transfer sequences, that is, the abstract structure. Fu et al. (2018), adopting three types of triplets in the training phase, confirmed that people can simultaneously and implicitly acquire both chunking and abstract knowledge in sequence learning. Consistently, Goschke and Bolte (2007), using a serial name task (SNT), found that subjects responded more quickly when the objects were in a repeating category sequence than when they were in a random category sequence, indicating learning of the category sequence. Kemeny and Meier (2016), using a task sequence learning (TSL) paradigm, demonstrated that people can implicitly acquire abstract conception representation in sequence learning.

However, these findings seem inconsistent to some previous studies that suggested that abstract structure could be acquired only in explicit learning conditions (Shanks and St John, 1994; Dominey and Jeannerod, 1997; Gomez, 1997; Dominey et al., 1998; Boyer et al., 2005). For example, Dominey et al. (1998) demonstrated that surface structures can be acquired by either implicit or explicit learners, but learning abstract structures could occur only for explicit learners. Boyer et al. (2005) also suggested that the core mechanism involved in sequence learning is statistical in nature and genuine rule-based knowledge is necessarily conscious. Nonetheless, more studies have recently provided new evidence that abstract relationships or concepts can be processed without awareness (Gross and Greene, 2007; Dienes et al., 2012; Lin and Murray, 2013; Tanaka and Watanabe, 2014a, b, 2015; Huang et al., 2017; Ling et al., 2018).

Therefore, to further explore how abstract structures can be implicitly acquired in sequence learning, a new computational model is proposed that provides a possible interpretation about the mechanism of implicit sequence learning. As in Dominey et al. (1998), the surface structure is defined as the straightforward serial order of sequence elements, whereas the abstract structure is defined as the relationship between repeating sequence elements in the present study. As the ability to abstract rules is an important component of higher cognition (Wallis et al., 2001), the investigation of how abstract structures can be acquired unconsciously would help understand how the human brain extends specific experience to general situations that are central to human intelligence.

### Computational Models for Sequence Learning

Computational models play a central role in the investigation of the nature of what is learned in implicit learning (Cleeremans and Dienes, 2008). The neural network is one of the most influential methods in computational models of implicit learning. A number of computational models using neural networks have been proposed for sequence learning (Cleeremans and McClelland, 1991; Cleeremans et al., 1998; Dominey et al., 1998; Sun et al., 2005; Cleeremans and Dienes, 2008).

**FIGURE 1:**
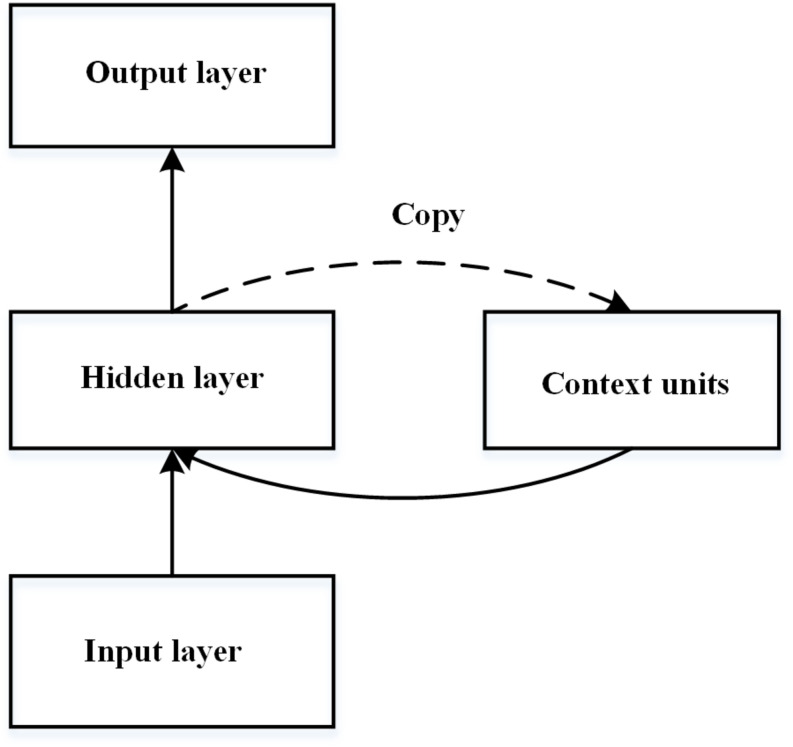
Schematic diagram of the simple recurrent network introduced by Elman (1990).

The simple recurrent network (SRN), first introduced by Elman (1990), is one of the most widely used neural network models of implicit learning (see Figure 1). The SRN is a three-layered connectionist neural network that consists of input, hidden, and output layers. It is trained to predict the next stimulus based on the current input and the previous stimuli. The prediction ability of the SRN stems primarily from the extra set of context units that contain a copy of the network’s pattern of activity. Cleeremans and McClelland (1991) first adopted the SRN model to imitate RT performance in the SRT task, in which the training sequence was generated by a finite-state grammar. The learned representation of the network is considered to be at a level of abstractness between exemplars and the finite state grammar (Cleeremans and Dienes, 2008) and is very close to the abstract representation of grammar (Cleeremans, 1993). However, the standard SRN is criticised because the learned knowledge is highly inflexible and domain dependent (Marcus, 2001). That is, the knowledge embedded in the connection weights is related to particular letters and no previous learning would be relevant when the new sets of input units are activated.

A dual process model is proposed to investigate how abstract structures can be learned independent of the acquisition of surface structure in sequence learning (Dominey et al., 1998). The dual process model assumes that two disassociate processes are involved in learning surface structures and abstract structures of sensorimotor sequences. The surface structure is learned based on an SRN model, whereas the abstract structure is learned dependent on short-term memory (STM), which encodes 7 ± 2 previous responses, and a recognition mechanism, which detects repeating elements between current responses and previous responses. The relevant behavioural and simulation results demonstrated that both surface and abstract structures can be captured by this dual process model, but abstract structures can be learned only explicitly. However, this is inconsistent with recent findings that people can implicitly acquire abstract structures (Lin and Murray, 2013; Tanaka and Watanabe, 2014a, b, 2015; Huang et al., 2017; Fu et al., 2018; Ling et al., 2018).

An augmented SRN is proposed to explore how the acquired knowledge of an artificial grammar can be transferred from one domain to another (Dienes et al., 1999). In the augmented SRN, an extra encoding layer between the input and hidden units of the standard SRN is added. This layer can provide an abstract recording of domain-dependent coding, which allows the acquired knowledge to be transferred from one domain to another. In the training phase, this model is trained similar to a standard SRN. After training, the core weights from the encoding layer to hidden units and from context units to hidden units are frozen and the network needs to learn the mappings to and from the abstract encoding formed by the SRN. Thus, the augmented SRN extends the function of the simple SRN by allowing the model to transfer its knowledge of an artificial grammar across domains without feedback. Nonetheless, in the augmented SRN, the frozen core wights might reflect learning of both abstract structures and surface structures of the trained domain. Of course, the learned abstract structures could be transferred to the other domains, but the learned surface structures might not. Moreover, it seems that the mappings between the trained and untrained stimuli in one domain are not necessary for the transfer processes. Thus, one may expect that the acquisition of abstract structures and surface structures might be embedded in different core weights in different learning systems. Also, the acquired knowledge of the trained stimuli can be transferred to new stimuli as long as they have the same abstract structure whenever they were in the same or different domains.

To further investigate how abstract structures can be obtained implicitly during sequence learning, we proposed a dual simple recurrent network (DSRN), in which chunking and abstract structures can be learned independently through a surface SRN and an abstract SRN. The surface SRN encodes the surface properties of stimuli and learns to predict the surface properties of the next stimulus, whereas the abstract SRN encodes the abstract properties of the stimuli and learns to predict the abstract properties of the next stimulus. We assume that either the abstract SRN or the surface SRN can learn implicitly, and the conscious status of its acquired knowledge is dependent on the particular condition under which they are trained. A response layer integrates the outputs of both SRNs to make the final prediction. We assume that the contribution weights of the abstract SRN and the surface SRN in the response layer are complementary and the sum of the two weights is always equal to 1, as the two SRNs contribute to the task performance at the same time but in different ways.

### A Dual Simple Recurrent Network (DSRN) for Sequence Learning

**FIGURE 2:**
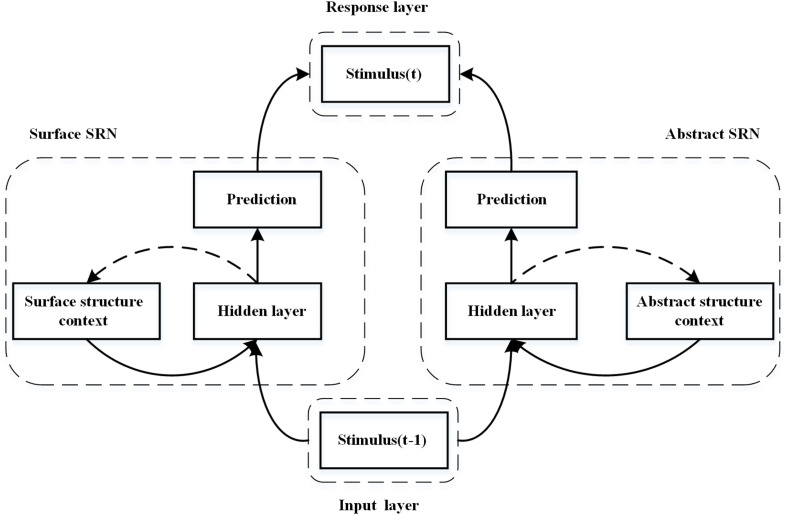
Schematic diagram of the dual simple recurrent network (DSRN). The left SRN encodes the sequence context and learns to predict the next stimuli, whereas the right SRN encodes the abstract structures in the stimuli sequence and learns to predict the abstract structure of the next stimulus. The predictions of the two SRNs are integrated in a response layer.

Figure 2 shows a schematic diagram of the proposed DSRN for sequence learning (Fu et al., 2015). The DSRN consists of two SRNs: a surface SRN learning surface structures of the stimulus sequence, and an abstract SRN learning abstract structures of the stimulus sequence.

The dynamic functions of each SRN are formulated as:

(1) $$\left\{ \begin{array}{c} a_{k}\,\left(t\right)=f_{k}\,\left(n\,e\,t_{k}\,\left(t\right)\right)\;\\ n\,e\,t_{k}\,\left(t\right)=\sum_{j}w_{k\,j}\,a_{j}\,\left(t\right)\;\\ a_{j}\,\left(t\right)=f_{j}\,\left(n\,e\,t_{j}\,\left(t\right)\right)\;\\ n\,e\,t_{j}\,\left(t\right)=\sum_{l}w_{j\,l}\,a_{l}\,\left(t-1\right)+\sum_{i}w_{j\,i}\,x_{i}\,\left(t-1\right) \end{array}\right.$$

where *a*_*k*_(*t*) is activation of the *k*-th unit in the output layer at time *t*, *a*_*j*_(*t*) is activation of the *j*-th unit in the hidden layer at time *t*, *a*_*l*_(*t*−1) is activation of the *l*-th unit at time *t-1*, *x*_*i*_(*t*−1) is activation of the *i*-th external input at time *t-1*, *f* is the activation function, and *w* is the connection weight. The sigmoid function is adopted as the activation function, $f\,\left(x\right)=\frac{1}{1+e\,x\,p^{-x}}$.

In each trial, a stimulus was presented to the DSRN model at time *t* and the model predicted the next stimulus based on the current stimulus and the previous inner states. The network’s output was compared to the actual stimulus and the error was back-propagated to adjust the connection weights (Hinton, 1986).

### Learning Mechanism in the DSRN

*Learning the* s*urface structure of SOC sequences*: In most recent studies, the only difference between training and transfer SOC sequences was the surface structure. For example, 3-4 was followed by a 2 in SOC1 but by a 1 in SOC2. At each time step, the current element in a sequence was presented as an input of the surface SRN and then produced a prediction in the output layer. Driven by the expected output, which is the next element in the same sequence, the surface SRN can copy the serial order of the surface structure in the context units. Due to the network’s recurrent connection architecture and adaptive inner representation, the surface SRN can learn the surface structure through training, e.g., the straightforward serial order of sequence elements. That is, the model responds to training stimuli more quickly and accurately than transfer stimuli.

*Learning the abstract structure of SOC sequences*: As in Dominey et al. (1998), the abstract structure is defined as the relationship between the repeating sequence elements. Although triplets from training and transfer SOC sequences had different surface structures, they shared the same abstract structure. That is, although a triplet from the training sequence (for example, 3-4-2) is different from the corresponding triplet from the transfer sequence (for example, a 3-4-1), they have a common abstract structure, that is, A-B-C. The two triplets were different from the triplet from neither training nor transfer sequences (for example, 3-4-3), of which the abstract structure can be considered as A-B-A. To obtain a representation of the abstract structure, the abstract SRN encoded the abstract property of the current stimulus and previous stimuli, e.g., the relationship between repeating sequence elements. As the surface SRN, the abstract SRN can learn the abstract structure and predict the abstract property of the next stimulus with training.

*Response selection*: The local representation was adopted in both the input and response layers. Assume that the learning materials were produced by combining and repeating any *m* elements, and each unit in the input or response layer corresponded to one of the *m* stimuli. Each unit of the response layer corresponded to one of the *m* possible responses and received only outputs of corresponding units in the output layer in both surface and abstract SRNs. For instance, the first response unit was influenced by the first output unit of the surface and abstract SRNs. The connections from the output unit to the response unit was fixed and the relative contribution of the two SRNs was balanced by a constant parameter ρ. The activation of i-th the units in the response layer was computed as:

(2) $$a_{i}\,\left(t\right)=\rho \,a_{i}^{l}\,\left(t\right)+\left(1-\rho \right)\,a_{i}^{r}\,\left(t\right)$$

where $a_{i}^{l}$ and $a_{i}^{r}$ are activation of the *i*-th output unit in the surface and abstract SRNs, respectively, and ρ is the contribution weight of the surface SRN to the response layer. The activation of the response units was recorded and then normalised with Luce’s rule (Luce, 2005).

(3) $$a_{i}\,\left(t\right)=\frac{a_{i}\,\left(t\right)}{\sum_{j}a_{j}\,\left(t\right)}$$

As in Cleeremans and McClelland (1991), we simulated the performance of the SRT task based on two assumptions. First, the prediction task performed by the SRN represents preparation for the apparition of the next stimulus in human subjects. Second, the RT is inversely related to the activation of the output units corresponding to the element being responded to. The reciprocal of the activation in the response layer is the DSRN’s reaction time.

### Tailored DSRN for Simulations

In each simulation, an initialisation process with different random parameters was conducted firstly to make DSRN models familiar with the SOC sequences. After that, two phases as in human experiments were conducted: a training phase, during which an SRT task was performed, and a testing phase, during which an inclusion test and an exclusion test were included. In the training phase, the DSRN models were first initialised with the pretrained parameters before the SRT task. Then, one of the stimuli was presented as an input of both SRNs and the model needed to respond to this stimulus, and the “reaction time” computed according to the reciprocal of output was recorded. Each model was trained with the same number of trials as participants received in the SRT task in the training phase. After the training phase, each model completed two free-generation tests. Each generation test began with the presentation of two randomly selected stimuli and then the model needed to predict the next stimuli based on the first two. Under the inclusion test, the model needed to select the output of the most activated units as the response to the predicted stimulus. However, under the exclusion test, the model needed to select the output of the least activated units as the response to the predicted stimulus. When one of the responses was selected, it would be presented as the current stimulus to the DSRN, and the DSRN needed to predict the next one on the basis of the current one and its previous one. Each model was tested with same number of trials as participants received in each test in each condition.

*Local coding*. As in Cleeremans and McClelland (1991), the local representation was used in both input and output units that encoded each possible sequence element. Each stimulus was represented as an m-bit vector (that is, *m* types of stimuli) and only one was activated as 1 and the others as 0. For example, if each stimulus was represented as a 4-bit vector, then stimulus 2 was represented as [0, 1, 0, 0]^T^. The abstract SRN had the same encoding as the surface SRN for the input stimuli. For example, the input encoding for the triplets 3-4-2 and 3-4-3 were both 0010-0001-0100 and 0010-0001-0010 in the two SRNs. The differences between surface and abstract SRNs were in their different output encodings. For the surface SRN, its output and input encodings were identical to any stimuli. However, for the abstract SRN, its output and input encodings were conceptually different in that there were only two types of the output encodings for any one stimulus, which was determined by the previous two stimuli. For example, the output encodings for the third stimulus of the triplets 3-4-3 and 3-4-2 in the abstract SRN were 0010 and 0110, respectively, while the output encodings for the same stimulus in the surface SRN were still 0010-0001-0100 and 0010-0001-0010.

*The SRT task in the training phase*. During each trial of human experiment, a stimulus appeared at one of four locations on a computer screen and the participants were asked to respond to each stimulus by pressing one corresponding key on the keyboard. Similarly, in the simulation experiment, each stimulus was presented to the network and the network needed to prepare for the response to the stimulus. As the prediction of the properties of the next stimulus was based on the preceding two stimuli, the DSRN predicted only from the third to the last one in each block. The prediction and learning processes are as follows:

• Prediction. Using Equations (1), (2), and (3), the DSRN is activated when the current stimulus is presented to the network. As in Cleeremans and McClelland (1991), we assumed that (1) the normalised activation of the response unit represents the response tendencies, and (2) there is a linear reduction in the RT proportional to the relative strength of the unit corresponding to the correct response. Thus, the activation of the response unit is recorded and the reciprocal of this activation is taken as the reaction time.
• Learning. After prediction, the back-propagation process immediately occurs in both SRNs. Error information is computed as how much the real output matches the expected output (Rumelhart et al., 1986). During the back-propagation phase, there is a key difference between the surface SRN and abstract SRN in the error information. The target activation of the expected output units is set to 1 and those of other units are set to 0. The surface SRN aims to learn the surface structure, and its expected output is the surface property of the next stimulus, whereas the abstract SRN aims to learn the abstract structure, and its expected output is the abstract property of the next stimulus.

*The free-generation task in the test phase*. To simulate generation performance, we use the output of the DSRN as a series of possible responses. One of the responses is selected based on the activation level of each response unit. The same operation is done for each trial in both inclusion and exclusion tests. However, the response selection is different between inclusion and exclusion tests. Under the inclusion test, if the output of the most activated units is close to the second one, the next input is randomly chosen from these two units; otherwise, it corresponds to the most activated output units. Under the exclusion test, this particular response is excluded and the next stimulus is chosen from the others.

*The method and parameters*. To simulate the experimental situation, the surface SRN and the abstract SRN have the same architecture of five hidden units. The response layer has the same number of units as the output layer. With different randomly initialised connections, we can generate many different DSRN models that allow us to conduct the similar analysis as in human experiment. In the simulation, all of the weights in the DSRN model are first initialised with small random values that are sampled from a uniform distribution on the open interval (0, 1). And then, the DSRN models are pretrained to be familiar with the SOC sequences. Using these generated models, the surface and abstract structure can be learned in the SRT task and then used to predict the next stimulus in the generation task. Different simulation of training and testing tasks has a different group of free parameters. The free parameters include the number of units in the hidden layer, the number of epochs for pre-training, the learning rate, the momentum, and ρ, i.e., the contribution weight of the surface SRN in the response layer. To be noted, as we assume that the sum of the contribution weights of the two SRNs is always equal to 1, if the contribution weight of the surface SRN, i.e., ρ, is determined, the contribution weight of the abstract SRN is also determined. Thus, in each simulation, we reported only the value of ρ, and the contribution weight of the abstract SRN can be calculated by 1 minus ρ. Further, as the contribution weight of each SRN is different for different tasks, ρ values are first tested to determine the best-fit one for each condition in each simulation. More details about the free parameters are given in each simulation.

## Simulation 1

Experiment 1 in Fu et al. (2008) adopted a deterministic sequence to explore whether people can implicitly acquire the SOC sequence in sequence learning and whether reward can influence performance of the free-generation test. The results revealed that people can learn the SOC structure implicitly and reward influences the performance of the exclusion generation test. In Simulation 1, we aimed to investigate whether the DSRN model can account for human performance in both the SRT and generation tasks during the experiment.

### Materials and Settings

**Materials:** Two SOC sequences (SOC1 = 3-4-2-3-1-2-1-4-3-2-4-1 and SOC2 = 3-4-1-2-4-3-1-4-2-1-3-2) were used in Simulation 1. The sequences were balanced for location frequency (each location occurred three times), transition frequency (each possible transition from one location to another that occurred once), reversal (for example 1-2-1) frequency (one in each sequence), repetitions (no repetitions in either sequence), and rate of full coverage (see Reed and Johnson, 1994). The difference between the sequences is in their second-order conditional structure. For example, 3-4 was followed only by a 2 in SOC1 but only by a 1 in SOC2.

**Human experiment:** In Experiment 1 in Fu et al. (2008), a deterministic SOC sequence was used in the SRT task. Keys D, F, J, and K corresponded to locations 1, 2, 3, and 4, respectively. Fifty-six undergraduate students (24 males, 32 females) took part in the experiment. They were randomly assigned to the no-reward or reward groups (*n* = 28, per group). The participants were instructed to respond as quickly and as accurately as possible by pressing one of the corresponding keys on the keyboard. After the training phase, the participants were informed that the targets had followed a regular repeating sequence, in which the stimulus locations were determined by the previous two. In the free-generation test, the participants were required to generate a sequence of 96 trials under both inclusion and exclusion instructions. The participants in the reward condition were informed that they would receive additional money for good performance before the generation test while the participants in the no-reward condition were not. The RT results indicated that the participants learned the second-order conditional structure, and the participants in the two groups learned equally about the sequential structure of the materials. Importantly, the reward significantly affected the exclusion performance in the free-generation test.

**Simulation settings:** In Simulation 1, 56 DSRN models were randomly divided into reward and no-reward conditions as in the human experiments. In the SRT task, all of the models in the reward and no-reward conditions were initialised with different random parameters. Each model was pre-trained with 2,500 randomly generated stimulus series as the key press training in the human experiments. Then, in the training phase of the SRT task, DSRN models were exposed to a series of four-choice RT tasks in both no-reward and reward conditions. There were 15 blocks in the training phase as in the human experiments. Each block consisted of 98 trials and began at a random point in one of the two SOC sequences. For counter balancing, half of the models in each condition were trained with SOC1 and half were trained with SOC2. Blocks from 1 to 12 and 14 to 15 consisted of the training sequence, while Block 13 consisted of the transfer sequence. In the test phase, there were an inclusion test and an exclusion test. In each test, two elements of the SOC sequence were first randomly presented to the trained DSRN model as in the training and then the model was required to generate a sequence of 96 trials. The DSRNs were only forbidden from generating the same element twice or more in a row under inclusion and exclusion tests. To determine the best-fit value of ρ, i.e., the best contribution weight of the surface SRN, we tested the fit of the model performance to human performance when ρ = [0, 0.1, 0.2, 0.3 0.4, 0.5, 0.6, 0.7, 0.8, 0.9, 1.0] in each condition of each task. When ρ = 0, it means that only the abstract SRN contributes to the task; When ρ = 1, it means that only the surface SRN contributes to the task. We found that when ρ was set at 0.8, the DSRN fitted the human performance the best in the SRT task. Moreover, when ρ was set at 0.7 for the reward condition and 0.8 for the no-reward condition in the generation task, the model performance could best reflect the difference between the reward and no-reward conditions.

### Simulation Results

**FIGURE 3:**
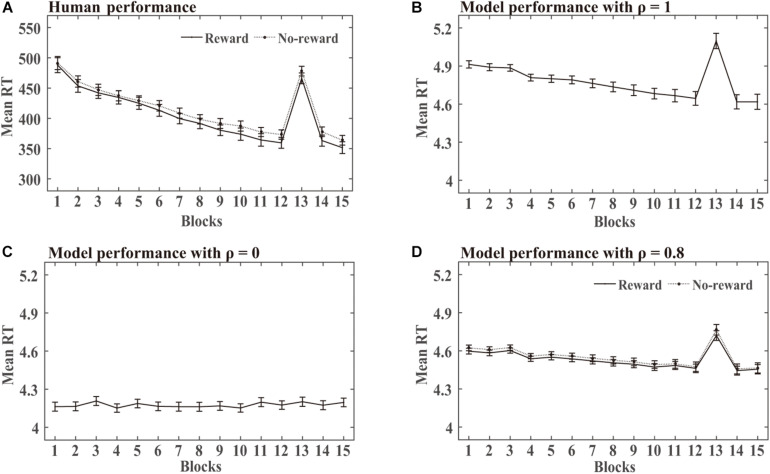
Human and model performance in the SRT task in Simulation 1. **(A)** Mean RTs for human participants in the SRT task. **(B–D)** Mean RTs for models with different ρ values in the SRT task.

**SRT task:** The statistical analysis of the mean RT in this simulation was similar to the human experiments. DSRN models trained with SOC1 and SOC2 were combined in each condition. Mean RT analyses were conducted for correct responses across 15 blocks. RTs for the first two targets of each block were excluded because they could not be predicted. Figure 3A shows the human RT performance in each condition. The RTs decreased from blocks 1 to 12, increased obviously in block 13, and then returned to lower level in blocks 14 and 15. There was no detectable difference between the two incentive groups in the training phase. Figures 3B–D illustrate the performance in the training phase by the DSRN models with different ρ values. The ρ value represented the relative contribution of the surface SRN. For ρ = 0, the performance was completely contributed by the activation of the abstract SRN; for ρ = 1, the performance was completely contributed by the activation of the surface SRN. The simulation results revealed that the RTs increased dramatically in block 13 for ρ = 1 but there was no marked increase for ρ = 0. The DSRN fit the human performance the best for ρ = 0.8, and thus the DSRN model with ρ = 0.8 was used to simulate the RT data in the training phase in both conditions.

For model performance, an ANOVA on the RTs with incentives (rewards vs no-rewards) as a “between-subjects” variable and blocks (15 levels) as a “within-subjects” variable revealed only a significant effect of the block, *F* (1.17, 62.94) = 129.48, *p* < 0.001, and η^2^_p_ = 0.71. That is, there was no detectable difference between the two incentive groups in the training phase. To examine the transfer effects, an ANOVA on the RTs with incentive as a “between-subjects” variable and blocks (transfer block 13 vs the average of blocks 12 and 14) as a “within-subjects” variable revealed only a block effect, *F* (1, 54) = 132.19, *p* < 0.001, and η^2^_p_ = 0.71. That is, the DSRN models in the two incentive conditions learned the difference between the training and transfer SOC sequences.

**FIGURE 4:**
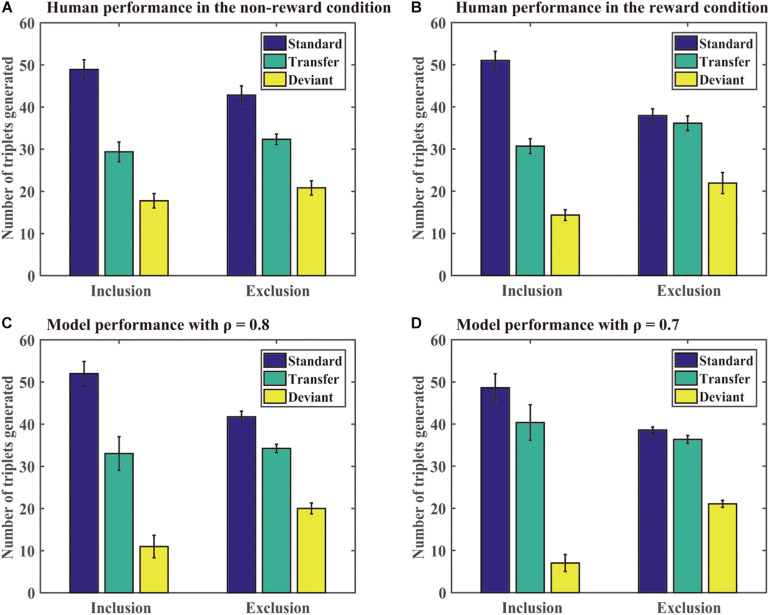
Human and model performance in the generation tests in Simulation 1. **(A)** The number of triplets generated by human participants in the no-reward condition. **(B)** The number of triplets generated by human participants in the reward condition. **(C)** The number of triplets generated by models in the no-reward condition. **(D)** The number of triplets generated by models in the reward condition.

**Generation task:** The DSRN models generated sequences of 96 trials under the inclusion or exclusion tests. We computed the number of each type of triplets generated in each test under each condition. A standard triplet was a triplet that was part of the training sequence, a transfer triplet was a triplet that was part of the transfer sequence, and a deviant triplet was a triplet that was from neither the training nor transfer sequence. Figure 4 shows the number of different triplets generated in human experiments and Simulation 1. If the training sequence was implicitly learned, one would expect no significant differences between the number of standard triplets under inclusion and exclusion or more standard than transfer triplets generated under the exclusion test; otherwise, the training sequence was explicitly learned. The DSRNs fit the human performance in the reward and no-reward conditions the best for ρ = 0.7 and ρ = 0.8, respectively, thus the DSRN models with ρ = 0.7 and ρ = 0.8 were used to simulate the generation data in the test phase in the two conditions.

We first compared the number of standard triplets generated under inclusion and exclusion instructions in the two conditions. An ANOVA with an incentive (no-reward vs reward) as a “between-subjects” variable and instructions (inclusion vs exclusion) as a “within-subjects” variable was conducted. It revealed only a significant instruction effect, *F* (1, 54) = 37.50, *p* < 0.001, and η^2^_p_ = 0.41, indicating that more standard triplets were generated under inclusion than exclusion for both the reward and no-reward groups.

We further compared the number of standard and transfer triplets generated under the exclusion test. An ANOVA with an incentive (no-reward vs reward) as a “between-subjects” variable and type of triplets (standard vs transfer) as a “within-subjects” variable was conducted. It revealed a significant effect of the triplet type, *F* (1, 54) = 7.77, *p* < 0.01, and η^2^_p_ = 0.13, and a significant triplet type by incentive interaction, *F* (1, 54) = 10.95, *p* < 0.01, and η^2^_p_ = 0.17. Simple effects of the triplet type for each incentive condition demonstrated that there was an effect of the triplet type in the no-reward condition (*p* < 0.001), but not for the reward condition (*p* = 71). The results suggested that the DSRN models with ρ = 0.7 in the reward condition were able to withhold their activation under the exclusion task, but the models with ρ = 0.8 in the no-reward condition could not.

Finally, we compared deviant triplets under inclusion and exclusion tests in each condition. An ANOVA on deviant triplets with incentive (no-reward vs reward) as a “between-subjects” variable and instructions (inclusion vs exclusion) as a “within-subjects” variable revealed only a significant instruction effect, *F* (1, 54) = 47.08, *p* < 0.001, and η^2^_p_ = 0.47. The results indicated that the DSRN models in both conditions generated greater deviant triplets under exclusion than inclusion.

### Comparing Model Performance With Human Performance

From Figures 3, 4, it can be seen that the DSRN models can simulate human performance very well. To provide a quantitative assessment of the fit of the model to human performance, the Pearson correlation coefficients between human participants and the corresponding DSRN models were taken as linear fits for the SRT and generation performance separately. In Simulation 1, the model accounted for approximately 89.0% of the variance in the SRT task and approximately 97.7% in the generation task.

In the SRT task, when the DSRN model was completely contributed by the activation of the abstract SRN (that is, ρ = 0), no significant differences were detected between transfer block 13 and its neighbouring training blocks; when the model was completely contributed by the activation of the surface SRN, more slow responses were observed for transfer block 13 than its neighbouring training blocks. The results indicated that block 13 was different from the other blocks for the chunking SRN, but was similar to the other blocks for the abstract SRN. When ρ = 0.8, the model accounted for the RT performance in the SRT task the best. The results indicated that both the chunking and abstract processes contributed to sequence learning in human performance.

In the generation task, different ρ values were used to simulate human generation performance under the no-reward and reward conditions. Interestingly, we found that manipulating the contribution of each SRN (that is, the ρ value), the DSRN could simulate the performance differences in the generation test between the no-reward and reward conditions. Specifically, under the exclusion test, the models generated more standard than transfer triplets for ρ = 0.8 but no significant differences between standard and transfer for ρ = 0.7. For participants, reward instructions in the generation tasks made participants express more conscious knowledge. Correspondingly, for models, it was the increase of the contribution of the abstract SRN from 0.2 (i.e., 1 minus 0.8) to 0.3 (i.e., 1 minus 0.7) that led the models to express more conscious knowledge in the generation tasks. The results suggested that manipulating the contribution of surface and abstract SRNs can mediate the expression of conscious knowledge in the generation task. This might be because that the number of the abstract structures was smaller (only two types) compared with the number of the surface structures (36 ones), the abstract structures should access consciousness earlier than the chunking knowledge.

## Simulation 2

Experiment 3 in Fu et al. (2008) used a probable SOC sequence to explore whether the amount of training (6 vs 15 blocks) can influence the conscious status of the acquired knowledge in sequence learning. The results revealed that people can acquire chunking and abstract knowledge in sequence learning and implicit or unconscious knowledge was detectable given a shorter rather than longer period of training. In Simulation 2, we aimed to investigate whether the DSRN model can account for human performance in the probable sequence learning in that experiment.

### Materials and Settings

**Materials:** The deterministic SOC sequence in Simulation 1 can be broken down into 12 sequential chunks with three locations or triplets (for example, SOC1 can be broken down into triplets 3-4-2, 4-2-3, 2-3-1, and so on; and SOC2 can be broken down into 3-4-1, 4-1-2, 1-2-4, and so on). In each triplet, the third location was completely determined by the previous two locations. In Simulation 2, we adopted the probabilistic sequences as in Experiment 3 in Fu et al. (2008), in which the stimuli followed the training SOC sequence with a probability of.875 and the transfer SOC sequence with a probability of.125.

**Human experiment:** In Experiment 3 in Fu et al. (2008), the participants were assigned to two conditions (6 block vs 15 block). Forty-eight undergraduate students (22 males, 26 females) took part in the experiment. They were randomly assigned to two groups (6-block, *n* = 24; 15-block, *n* = 24). The training phase adopted the probabilistic sequences. The test phase was identical to the reward condition in Experiment 1 in Fu et al. (2008).

**Simulation settings:** Forty-eight DSRN models were assigned to two conditions (6 block vs 15 block) as in the human participants in Experiment 3 in Fu et al. (2008). Each model was initialised with different random parameters and pre-trained with 2,500 randomly generated stimulus series. Then the models under the 6-block condition were trained with six blocks and the models under the 15-block condition were trained with 15 blocks. Each block consisted of 98 trials with a probability of.875 from the training sequence and a probability of.125 from the transfer sequence. There was a total of 588 and 1,470 trials in the training phase depending on the condition. All the other settings about the DSRN are similar to Simulation 1.

### Simulation Results

**FIGURE 5:**
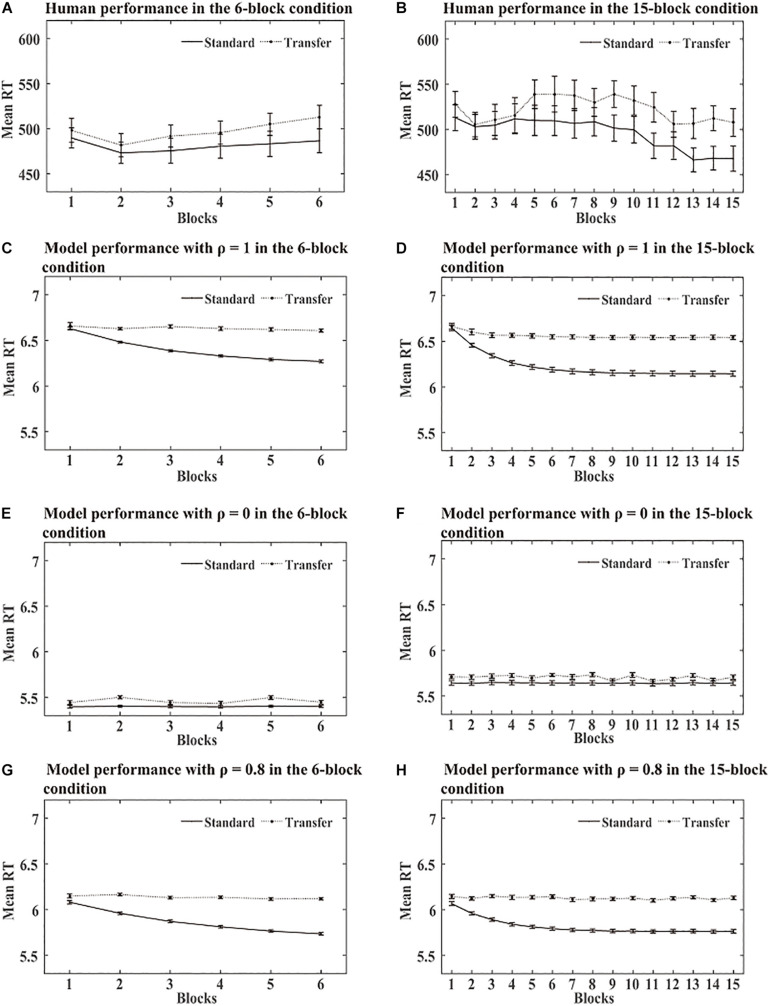
Human and model performance in the SRT task in Simulation 2. **(A)** Mean RTs for human participants in the 6-block group. **(B)** Mean RTs for human participants in the 15-block group. **(C,E,G)** Mean RTs for models with different ρ values in the 6-block condition. **(D,F,H)** Mean RTs for models with different ρ values in the 15-block condition.

**SRT task:** As a standard triplet was a triplet from the training sequence and a transfer triplet was a triplet from the transfer sequence, we computed RTs for standard and transfer triplets separately for each block in each condition. Figure 5 shows the RTs obtained over the training phase in the human and simulation experiments. The simulation results revealed no significant RT differences between the standard and transfer triplets for ρ = 0, while the RTs were much faster for the standard triplets than the transfer triplets for ρ = 1. As in Simulation 1, we found that the DSRN model fit the human performance the best for ρ = 0.8, and thus the DSRN with ρ = 0.8 was used to simulate the RT data in the training phase.

For the 6-block condition, an ANOVA on the RTs with triplets (standard vs transfer) and blocks (6 levels) as “within-subjects” variables revealed a significant effect of the triplets, *F* (1, 23) = 155.57, *p* < 0.001, and η^2^_p_ = 0.87, suggesting that the DSRN models responded more rapidly to the standard triplets than the transfer triplets. The main effect of the block was also significant, *F* (5, 115) = 117.40, *p* < 0.001, and η^2^_p_ = 0.84, indicating that the DSRN models responded to the targets more rapidly later in practise than earlier. The triplets by block interaction also reached significance, *F* (2.35, 54.15) = 75.60, *p* < 0.001, and η^2^_p_ = 0.77, indicating a greater triplet effect later in practise than earlier.

For the 15-block condition, a comparable ANOVA revealed a significant effect of the triplets, *F* (1, 23) = 139.53, *p* < 0.001, and η^2^_p_ = 0.86, suggesting that the DSRN models responded more rapidly to the standard triplets than the transfer triplets. The main effect of the block was significant, *F* (14, 322) = 75.45, *p* < 0.001, and η^2^_p_ = 0.77, and the triplets by block interaction also reached significance, *F* (14, 322) = 44.95, *p* < 0.001, and η^2^_p_ = 0.66, indicating a greater triplet effect later in practise than earlier.

**FIGURE 6:**
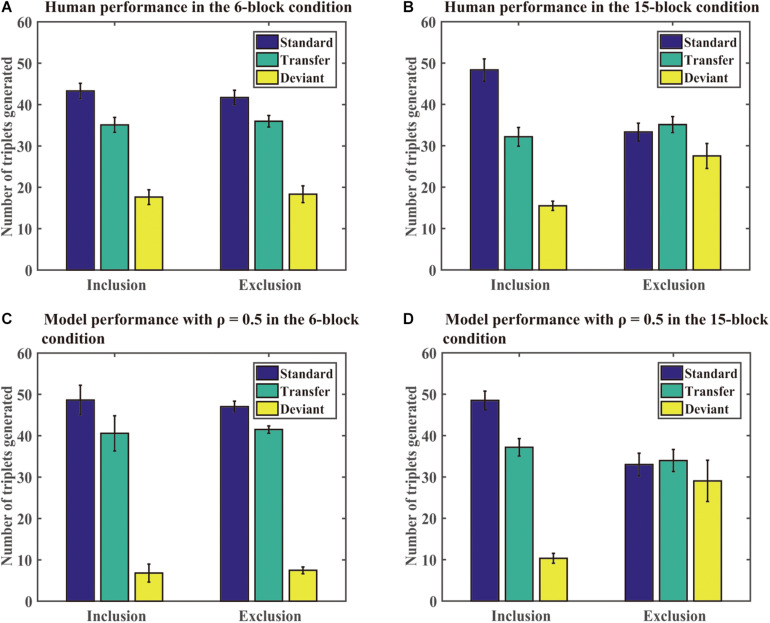
Human and model performance under inclusion and exclusion tests in Simulation 2. **(A)** The number of triplets generated by human participants in the 6-block condition. **(B)** The number of triplets generated by human participants in the 15-block condition. **(C)** The number of triplets generated by DSRN models in the 6-block condition. **(D)** The number of triplets generated by models in the 15-block condition.

**Generation task:** As in the SRT task, we found that the DSRN model fit the human performance the best for ρ = 0.5 in both the 6- and 15-block conditions, and thus the DSRN with ρ = 0.5 was used to simulate the generation data in the test phase. Figure 6 shows the mean number of triplets generated in each condition in the human and simulation experiments. As in Simulation 1, we first compared the proportion of standard triplets generated under inclusion and exclusion instructions in the two conditions. An ANOVA on the standard triplets with training (6 block vs 15 block) as a “between-subjects” variable and instructions (inclusion vs exclusion) as a “within-subjects” variable revealed a significant instruction effect, *F* (1, 46) = 11.54, *p* = 0.001, and η^2^_p_ = 0.20, and the training by instruction interaction was also significant, *F* (1, 46) = 7.66, *p* < 0.01, and η^2^_p_ = 0.14. Simple effects of instruction for each training condition showed that there were more standard triplets under inclusion than exclusion for the 15-block condition, *F* (1, 46) = 18.99 and *p* < 0.001, but not for the 6-block condition, *F* (1, 46) = 0.20 and *p* = 0.66. The results revealed that the DSRN models in the 15-block condition generated more standard triplets in inclusion than exclusion, but the DSRN models in the 6-block condition did not.

To compare the number of standard and transfer triplets generated under the exclusion test, an ANOVA with training (6 block vs 15 block) as a “between-subjects” variable and type of triplets (standard vs transfer) as a “within-subjects” variable was conducted. It revealed a significant effect of training, *F* (1, 46) = 18.27, *p* < 0.001, and η^2^_p_ = 0.28, and a triplet type by training interaction, *F* (1, 46) = 4.71, *p* < 0.05, and η^2^_p_ = 0.09. Simple effects of the type of each training condition revealed that there was an effect of type for the 6-block condition, *F* (1, 46) = 6.84 and *p* < 0.05, but not for the 15-block condition, *F* (1, 46) = 0.20 and *p* = 0.65. The results indicated that the DSRN models in the 6-block condition could generate more standard than transfer sequences under exclusion instructions, but the DSRN models in the 15-block condition could not.

To compare the number of deviant triplets under inclusion and exclusion instructions in each condition, an ANOVA on deviant triplets with training (6 block vs 15 block) as a “between-subjects” variable and instructions (inclusion vs exclusion) as a “within-subjects” variable was conducted. It revealed a significant effect of instructions, *F* (1, 46) = 11.81, *p* < 0.001, and η^2^_p_ = 0.20, and a significant effect of training, *F* (1, 46) = 20.01, *p* < 0.001, and η^2^_p_ = 0.30. The instruction by training interaction also reached significance, *F* (1, 46) = 10.24, *p* < 0.01, and η^2^_p_ = 0.18. Simple effects of the instructions for each training condition showed that there was an effect of instruction for the 15-block condition, *F* (1, 46) = 22.02 and *p* < 0.001, but not for the 6-block condition, *F* (1, 46) = 0.03 and *p* = 0.87. The results suggested that the DSRN models in the 15-block condition generated greater deviant triplets under exclusion than inclusion, but the DSRN models in the 6-block condition did not.

### Comparing Model Performance With Human Performance

A probabilistic sequence rather than a deterministic sequence was adopted in Simulation 2. Figures 5, 6 show that the DSRN models could be also fit to simulate human performance in probabilistic sequence learning. The model accounted for approximately 71.8% of the variance in the RT task and approximately 96.7% in the generation task.

In the SRT task, when the DSRN model was completely contributed by the activation of the abstract SRN (that is, ρ = 0), no significant differences were detected between the standard and transfer triplets in both training conditions; when the model was completely contributed by the activation of the surface SRN (that is, ρ = 1), RTs were faster for standard than transfer triplets in both conditions. As in Simulation 1, we found that the model accounted for the RT performance for ρ = 0.8 in the SRT task the best. The results indicated that the architecture of the DSRN model can illustrate not only learning of deterministic sequences but also the learning effect of probable sequences.

For the generation task, the same ρ values (that is, ρ = 0.5) were used to simulate human generation performance in the 6-block and 15-block training conditions. On the one hand, the DSRN models with six training blocks could not generate more standard triplets under inclusion than exclusion tests, but could generate more standard than transfer triplets under the exclusion test, indicating that the chunking structure of the training sequence was acquired implicitly. On the other hand, the DSRN models with 15 training blocks could generate more standard triplets under inclusion than exclusion test, but generated similar standard and transfer triplets under the exclusion test, suggesting that the chunking structure of the training sequence was acquired explicitly. Thus, the DSRN models successfully simulated how the amount of training influenced the conscious status of the acquired chunking knowledge in probabilistic sequence learning.

Moreover, the DSRN models in the 15-block condition generated fewer deviant triplets under inclusion than exclusion, while the DSRN models in the 6-block condition generated similar deviant triplets under inclusion and exclusion. As the standard and transfer triplets shared the same abstract structure whereas the deviant triplets had a different abstract structure, the results indicated that the DSRN models could control the expression of knowledge about the abstract structure in the 15-block condition, but could not in the 6-block condition. That is, the abstract structure of the training sequence was acquired explicitly in the 15-block condition, but acquired implicitly in the 6-block condition. The results confirmed that unconscious knowledge was detectable given a shorter rather than longer training period.

## Simulation 3

Fu et al. (2018) used a probable SOC sequence that included three types of stimuli in the training phase to further explore how associated or chunking learning can dissociate from rule or abstract learning. The results revealed that people can simultaneously acquire knowledge about chunking and abstract structures, and the ability to control under inclusion and exclusion tests was mainly based on knowledge about abstract structures rather than concrete chunks. As Fu et al. (2018) used a trial-by-trial generation task rather than a free-generation task, we first ran a new human experiment in Simulation 3, in which three types of stimuli were adopted in the training phase and the free-generation task was used in the test phase. Then we investigated whether the DSRN models could account for human performance in the new experiment.

### Materials and Settings

**Materials:** In Simulation 3, we adopted the probabilistic sequences as in Experiment 3 in Fu et al. (2018). There were three types of stimuli in the training phase: standard stimuli following the training SOC sequence with a probability of 0.833, transfer stimuli following the transfer SOC sequence with a probability of 0.083, and deviant stimuli following neither the training nor transfer SOC sequence with a probability of 0.083.

**Human experiment:** Twenty-six university students (11 females, 15 males; mean age = 22.12 years, SD = 2.64 years) voluntarily took part in the experiment. They were paid for their attendance. None of them had previously taken part in any implicit learning experiment. All of them had normal or corrected-to-normal vision. This experiment was approved by the committee for the protection of subjects at the Institute of Psychology, Chinese Academy of Sciences. In the training phase, each participant was trained on 6 blocks. Each block consisted of 146 trials, for a total of 876 trials. The test phase was similar to the reward condition in Experiment 1 in Fu et al. (2008). The data from two participants (two males) were excluded because their accuracy in the SRT task was below 90%.

**Simulation settings:** Twenty-four DSRN models were used to simulate human performance. Each model was randomly initialised and pre-trained with 1,000 randomly generated stimuli. All of the models were trained on 6 blocks as in the human experiment. Half of the models were trained with SOC1, and half were trained with SOC2. As in Simulations 1 and 2, when the DSRN was ρ = 0.8, the model performance was best fit for human performance in both the SRT task and the generation task. Thus, we used ρ = 0.8 to simulate the RT data and the generation data in Simulation 3. All other settings about the DSRN were similar to Simulation 1.

### Human Experimental Results

**FIGURE 7:**
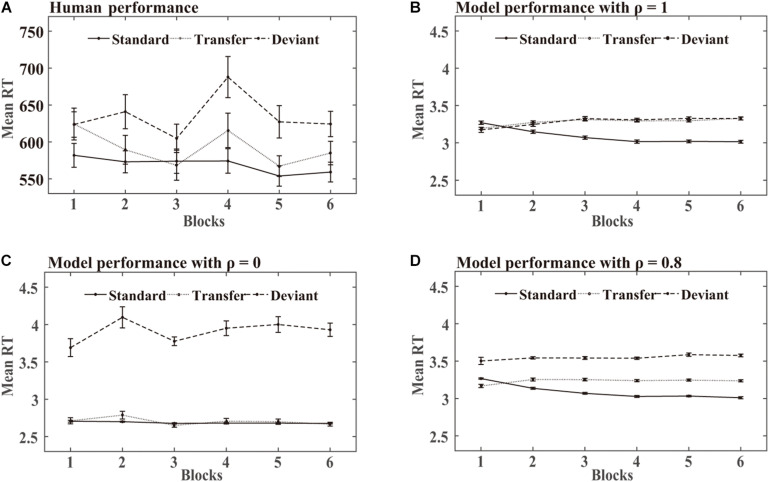
Human and model performance in the SRT task in Simulation 2. **(A)** Mean RTs for human participants. **(B–D)** Mean RTs for models with different ρ values.

**SRT task:** Figure 7 shows the mean RTs obtained over the training phase in the human experiments and DSRN simulations. For human performance, an ANOVA on the RTs with stimuli (standard vs transfer vs deviant) and blocks (6 levels) as within-subjects variables revealed a significant effect of the stimuli, *F* (2, 46) = 90.85, *p* < 0.001, and η^2^_p_ = 0.80. *Post hoc* analysis revealed that the participants responded faster to the standard than transfer stimuli (*p* < 0.001) and faster to the transfer than deviant stimuli (*p* < 0.001), indicating that the participants acquired knowledge about both chunking and abstract structures. The main block effect was also significant, *F* (5, 115) = 90.85, *p* = 0.001, and η^2^_p_ = 0.16, suggesting that the participants responded to the targets more rapidly later in practise than earlier. The stimuli by block interaction also reached significance, *F* (4.13, 94.89) = 3.09, *p* < 0.05, and η^2^_p_ = 0.12, indicating a greater stimuli effect later in practise than earlier.

**FIGURE 8:**
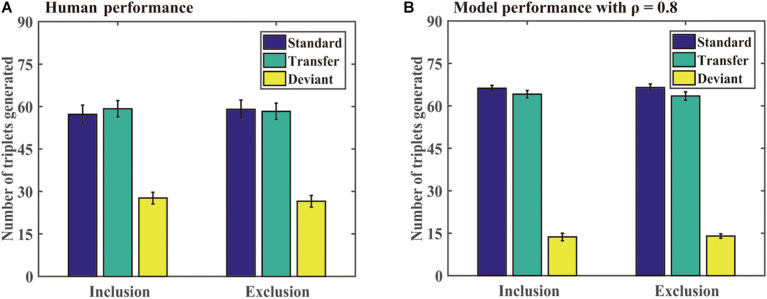
Human and model performance under inclusion and exclusion tests in Simulation 3. **(A)** The number of triplets generated by human participants. **(B)** The number of triplets generated by models.

**Generation task:** Figure 8 shows the number of different triplets generated by the participants and DSRNs in the generation task. For human performance, a paired-sample *t* test revealed that there were no significant differences in the standard triplets generated under inclusion and exclusion tests, *t* (23) = −0.57 and *p* = 0.57, and so were in the transfer and deviant triplets, *t* (23) = 0.26, *p* = 0.79, *t* (23) = 0.45, and *p* = 0.66, respectively. Moreover, under the exclusion test, the participants generated more standard than deviant triplets, *t* (23) = 9.10, *p* < 0.001, and *d* = 1.90, and more transfer than deviant triplets, *t* (23) = 8.36, *p* < 0.001, and *d* = 1.74, but there were no significant differences between the standard and transfer triplets, *t* (23) = 0.33 and *p* = 0.74. The results suggested that the participants implicitly acquired knowledge about chunking and abstract structures in the training phase.

### Simulation Results

In the simulation, when the DSRN was completely contributed by the activation of the surface SRN (that is, ρ = 1), the responses were faster for the standard than transfer triplets, but no significant differences were observed between the transfer and deviant triplets; when the DSRN was completely contributed by the activation of the abstract SRN (that is, ρ = 0), the responses were faster for the transfer than deviant triplets, but no significant differences were observed between the standard and transfer triplets.

**SRT task:** For the model performance in the SRT task, an ANOVA on the RTs with stimuli (standard vs transfer vs deviant) and blocks (6 levels) as “within-subjects” variables revealed a significant effect of stimuli, *F* (1.28, 29.41) = 324.28, *p* < 0.001, and η^2^_p_ = 0.93. *Post hoc* analysis revealed that the models responded faster to the standard than transfer stimuli (*p* < 0.001), and faster to the transfer than deviant stimuli (*p* < 0.001), indicating that they acquired knowledge about both chunking and abstract structures. The main effect of the blocks was not significant, *F* (1.93, 44.35) = 2.03, *p* = 0.15, and η^2^_p_ = 0.08. The stimuli by block interaction reached significance, *F* (4.60, 105.79) = 34.02, *p* < 0.001, and η^2^_p_ = 60, indicating a greater stimuli effect later in practise than earlier.

**Generation task:** For model performance in the generation task, a paired-sample *t* test revealed that there were no significant differences in the standard triplets generated under inclusion and exclusion tests, *t* (23) = −0.18 and *p* = 0.86, and no significant differences in the transfer and deviant triplets under inclusion and exclusion tests, *t* (23) = 0.35, *p* = 0.73, *t* (23) = −0.23, and *p* = 0.82, respectively. Moreover, under the exclusion test, the participants generated more standard than deviant triplets, *t* (23) = 36.22, *p* < 0.001, and *d* = 5.17, and more transfer than deviant triplets, *t* (23) = 24.81 and *p* < 0.001, and *d* = 5.17, but there were no significant differences between the standard and transfer triplets, *t* (23) = 1.15 and *p* = 0.26. The results confirmed that the two types of knowledge could be implicitly acquired.

### Comparing the DSRN Performance With Human Performance

In Simulation 3, a probabilistic sequence with three types of stimuli was adopted in the training phase in the human and simulation experiments. The participants and models both responded faster to the standard than transfer stimuli and faster to the transfer than deviant stimuli, confirming that the chunking and abstract structures could be acquired simultaneously in sequence learning. Moreover, the model accounted for approximately 77.1% of the variance in the RT task and approximately 99.7% in the generation task.

In the SRT task, the standard and transfer stimuli shared the same abstract structure, but one appeared with a high probability of 0.833 the other with a low probability of 0.083; the transfer and deviant stimuli both appeared with a low probability of 0.083, but had different abstract structures. The simulation results revealed that the surface SRN acquired the difference between the standard and transfer stimuli, that is, the chunking structure, while the abstract SRN learned the difference between the transfer and deviant stimuli, that is, the abstract structure. The models could successfully account for human performance only when both SRNs contributed to the models, providing new evidence that both the chunking and abstract processes contributed to sequence learning in human performance.

In the generation test, we found that the participants generated similar standard triplets under inclusion and exclusion tests, indicating that they could not control the expression of the standard triplets. That is, the chunking knowledge about the training sequence was acquired implicitly. Moreover, although the exclusion instructions asked the participants to generate the sequence rarely appeared in the training phase, the participants generated more standard and transfer triplets than deviant triplets, but no significant differences were observed between the standard and transfer triplets. On the one hand, the results indicated that the participants could not distinguish the standard from transfer stimuli, confirming that they implicitly acquired knowledge about the chunking structure. On the other hand, the results suggested that the participants could not inhibit generating more standard and transfer triplets than deviant triplets under exclusion, suggesting that they implicitly acquired knowledge about the abstract structure. Further, the DSRN models with ρ = 0.8 accounted very well for the human performance in the generation test. The results provided convergent evidence that both abstract learning and chunking learning can occur implicitly.

## General Discussion

The purpose of the current study was to investigate how people can acquire abstract knowledge in implicit sequence learning. To address this issue, we proposed a DSRN model that included a surface SRN encoding and predicting the surface properties of the stimuli, and an abstract SRN encoding and predicting the abstract properties of the stimuli in sequence learning. The simulation results in Simulations 1 and 2 showed that the DSRN model accounted for learning effects in both the RT and generation performance whenever the training sequence was a deterministic or probabilistic sequence. Moreover, manipulating the contribution weight of each SRN could also account for the contribution of conscious and unconscious processes in inclusion and exclusion tests under different conditions. The human performance results in Simulation 3 provided further evidence that people can simultaneously learn both chunking and abstract knowledge in implicit sequence learning, and the results of Simulation 3 confirmed that the DSRN model can account for how people acquire the two types of knowledge in implicit sequence learning.

### What Is Acquired in Implicit Sequence Learning?

Although many studies using artificial grammar learning paradigms provided evidence for the acquisition of abstract knowledge in implicit learning, fewer studies investigated abstract learning in the SRT task. This is partially because it is difficult to dissociate chunking or associative learning from abstract or rule learning in sequence learning. To solve this problem, three types of stimuli were adopted in the training phase in Simulation 3 as in Fu et al. (2018): standard and transfer stimuli, followed the same abstract structure but differed in the probability of occurrence, whereas the transfer and deviant stimuli occurred with the same low probability but differed in the abstract structure. The human results showed that the participants responded faster to the standard than transfer stimuli and faster to the transfer than deviant stimuli, indicating both chunking and abstract structures can be acquired in sequence learning. Importantly, the participants and models generated similar standard triplets under inclusion and exclusion and more standard and transfer triplets than deviant triplets under the exclusion test, suggesting that they implicitly acquired chunking and abstract knowledge. The results were consistent with previous findings that supported that people can acquire not only knowledge about the associations of specific stimuli but also the underlying deep structure in implicit sequence learning.

To illustrate how people can acquire sequence knowledge in sequence learning, we proposed a DSRN model that included a surface SRN learning the surface structure of stimuli and an abstract SRN learning the abstract structure of stimuli in sequence learning. The relative contribution of each SRN was mediated by a constant parameter ρ. Particularly, whenever the stimuli were presented, the abstract SRN would encode the abstract property of the current stimulus and previous stimuli, e.g., the relationship between repeating sequence elements. Thus, as long as the new stimuli have the same abstract structures with the trained stimuli, the acquired abstract knowledge can be transferred to the new stimuli to some extent. In this way, the DSRN model has no need to learn the mappings between the stimuli from different domains in the input and output layers as in the augmented SRN (Dienes et al., 1999). The proposed DSRN model was used to simulate learning effects in the SRT task in previous studies (Fu et al., 2008, 2018), in which the learning sequence was either deterministic or probabilistic. Across the three simulation experiments, the results provided clear evidence that the proposed DSRN model could account for human RT performance only when both the surface and abstract SRNs were involved in the SRT task, confirming that both the chunking and abstract processes contributed to sequence learning in human performance.

We believe that these results were principally consistent with the dual pathways hypothesis (Keele et al., 2003), which assumed that two learning systems underlie a sequence learning device: one learning system is multidimensional or abstract and builds associations between events from different dimensions or modalities, while the other system is unidimensional or concrete and associates non-categorised stimuli within dimensional modules. Kemeny and Meier (2016) provided the first empirical support for this hypothesis by demonstrating that multimodal sequence learning can occur implicitly. The present findings extend it by further demonstrating that not only the semantic category sequence (e.g., Implement-Plant-Animal-Plant-Implement-Animal) but also the other abstract sequence structure (e.g., A-B-C vs. A-B-A) can be acquired in implicit sequence learning.

### Relationship Between Abstract Knowledge and Consciousness

The results of Simulation 1 revealed that manipulating the contribution weight of each SRN could account for the effects of the reward on the generation performance, that is, the expressed conscious knowledge in the free-generation task. The results indicated that the contribution weight of each SRN might mediate the contribution of conscious and unconscious processes in inclusion and exclusion tests under different conditions. The findings provide a different and new perspective on the relationship between abstract knowledge and conscious awareness.

We found that adding the contribution of the abstract SRN rather than that of the surface SRN caused the models to express more conscious knowledge in the generation task in Simulation 1. That is, for models, it was the increase of the contribution of the abstract SRN from 0.2 to 0.3 that led the models to express more conscious knowledge in the generation tasks. This may have occurred because the abstract SRN acquired explicit knowledge and the surface SRN learned implicit knowledge. This was also consistent with the findings of Dominey et al. (1998), which illustrated that the rule was acquired only in explicit learning. However, our results revealed that abstract knowledge can be acquired implicitly with a short training phase. Specifically, the generation performance in Simulations 2 and 3 revealed that when the training phase included only six blocks, the participants and models could not control the generation of similar deviant triplets under inclusion and exclusion tests and more standard and transfer than the deviant triplets were generated under the exclusion test. The results provided clear evidence that abstract knowledge can be acquired implicitly.

Another possible explanation for this phenomenon might be that although both types of knowledge could be acquired implicitly, the knowledge acquired by the abstract SRN could be more easily accessible to consciousness than the knowledge acquired by the surface SRN. This is because generally there are more surface structures than abstract structures in implicit learning. For example, in the present study, there were 36 concrete triplets for chunking learning, but only two abstract structures for abstract learning. If we assume that all of the triplets were homogeneous, each abstract triplet was trained 17 times more often than each concrete triplet. As unconscious knowledge was detectable given a shorter rather than longer training period, the abstract structure should access consciousness earlier than chunk knowledge. In the other tasks with more complex abstract structures than the SRT task (for example, AGL tasks), the abstract structure might not be so easily available to consciousness. This is also supported by the view that explicit knowledge is in a way extracted from implicit knowledge and implicit knowledge always remains ahead of explicit knowledge (Bowers et al., 1990; Sun et al., 2005).

Moreover, the present findings also helped account for the inconsistent findings about abstract learning and consciousness. Although more recent studies have provided new evidence that abstract learning can occur unconsciously (Dienes et al., 2012; Kemeny and Meier, 2016; Huang et al., 2017; Ling et al., 2018), other studies supported that abstract knowledge can only be acquired consciously (Shanks and St John, 1994; Dominey et al., 1998; Boyer et al., 2005; Cleeremans and Destrebecqz, 2005). According to our findings, this might be because the former studies used relatively simple abstract structures while the latter studies used more complex structures. Further research can manipulate the complexity of the abstract structure to investigate whether acquired knowledge in implicit learning can be thought of as a point existing somewhere along the continuum from abstract representations to exemplar-based representations (Cleeremans and Destrebecqz, 2005).

In summary, our results provided convergent evidence that both surface and abstract structures can be acquired implicitly in sequence learning. The proposed DSRN model can account for how the two types of learning can occur simultaneously. Specifically, the simulation results revealed that the DSRN model can account not only for human learning effects in the SRT task but also how the conscious status of the expressed knowledge is influenced by different factors in the free-generation task. These findings extend the ability of the SRN model to learn and help understand how different types of knowledge can be acquired simultaneously in implicit sequence learning.

## Data Availability Statement

The raw data supporting the conclusions of this article will be made available by the authors, without undue reservation.

## Ethics Statement

The studies involving human participants were reviewed and approved by the Ethics Committee for the Protection of Subjects at the Institute of Psychology, Chinese Academy of Sciences. The patients/participants provided their written informed consent to participate in this study.

## Author Contributions

LW, YF, JW, and QF designed the experiments. LW, YF, QF, and XS performed the experiments and analysed the collecting data. LW, YF, QF, XF, LZ, and ZY wrote and revised the manuscript. All authors contributed to the article and approved the submitted version.

## Conflict of Interest

The authors declare that the research was conducted in the absence of any commercial or financial relationships that could be construed as a potential conflict of interest.
